# Design methodology for a confocal imaging system using an objective microlens array with an increased working distance

**DOI:** 10.1038/srep33278

**Published:** 2016-09-12

**Authors:** Woojae Choi, Ryung Shin, Jiseok Lim, Shinill Kang

**Affiliations:** 1 School of Mechanical Engineering, Yonsei University, Seoul, 03722, South Korea; 2 National Center for Optically-assisted Ultrahigh-precision Mechanical Systems, Yonsei University, Seoul, 03722, South Korea; 3 School of Mechanical Engineering, Yeungnam University, Gyeongsan, 38541, South Korea

## Abstract

In this study, a design methodology for a multi-optical probe confocal imaging system was developed. To develop an imaging system that has the required resolving power and imaging area, this study focused on a design methodology to create a scalable and easy-to-implement confocal imaging system. This system overcomes the limitations of the optical complexities of conventional multi-optical probe confocal imaging systems and the short working distance using a micro-objective lens module composed of two microlens arrays and a telecentric relay optical system. The micro-objective lens module was fabricated on a glass substrate using backside alignment photolithography and thermal reflow processes. To test the feasibility of the developed methodology, an optical system with a resolution of 1 μm/pixel using multi-optical probes with an array size of 10 × 10 was designed and constructed. The developed system provides a 1 mm × 1 mm field of view and a sample scanning range of 100 μm. The optical resolution was evaluated by conducting sample tests using a knife-edge detecting method. The measured lateral resolution of the system was 0.98 μm.

Micro/nano fabrication technologies are now highly integrated and scalable for application in semiconductor, display, and biological technologies^1^,^2^,^3^,^4^,^5^,^6^,^7^,^8^,^9^,^10^,^11^,^12^,^13^. As applications of micro/nano fabrication have expanded, demand for cost-effective, high-resolution, and large-area imaging technologies for inspecting the final structure have become more important to secure high manufacturing throughput^14^,^15^,^16^,^17^,^18^,^19^,^20^. In the case of the optical system currently in use for inspection, however, it is difficult to satisfy both the required resolution and the required imaging area^21^,^22^,^23^. There have been attempts to adopt a confocal optical system to overcome the limitations of the resolving power^24^,^25^,^26^. However, it is difficult to apply conventional confocal imaging systems to large-area inspection, mainly due to the low imaging throughput of confocal imaging systems. Various methods have been investigated to overcome these limitations by improving the imaging throughput of confocal imaging systems using multi-optical probes generated using a microlens array^27^,^28^,^29^. Using a microlens array as the objective lens of a confocal optical system allows this technique to overcome the mentioned limitations and provides additional advantages. First, when the microlens is used to obtain the optical information at the on-focus plane, the microlens is not affected by four Seidel aberrations: coma, astigmatism, field curvature, and distortion. The microlens is only affected by a spherical aberration. Second, large-scale applications become possible by expanding the dimensions of the array. Third, the microlens array can be fabricated at a low cost using a conventional photolithography process^13^,^30^.

Despite these advantages, the use of microlens arrays as an objective lens in a confocal imaging system cannot be put to practical use. This is mainly because the working distance, as determined by the focal length of the microlens, is too short. The array is, therefore, highly susceptible to damage from particulate contamination and microlens contact and to secondary scratch damage on the samples. Additionally, the implementation of a confocal imaging system using a microlens array as an objective lens requires a highly complex optical system that is not easily scalable.

This study focused on developing a design methodology for a scalable, easy-to-integrate confocal imaging system using an objective microlens array with an increased working distance. To test the feasibility of the design methodology, this array was used to construct a multi-optical probe confocal imaging system with an array size of 10 × 10. The developed system provides a field of view (FOV) of 1 mm × 1 mm with a sample scanning range of 100 μm, a resolution of 1-μm/pixel, and a working distance of 165.5 μm, which is greater than the maximum particulate contamination size possible during the manufacture of an actual display substrate^31^,^32^,^33^,^34^. The design methodology incorporates a micro-objective lens module (μOLM) composed of two microlens arrays, an objective microlens array (OMA), and an intermediate microlens array (IMA) integrated on either side of a glass substrate. Backside alignment photolithography and a thermal reflow process were applied to fabricate the μOLM. This module was combined with a conventional objective-side telecentric lens system to create a confocal imaging system. The point spread function (PSF) was measured using knife-edge detection to verify the feasibility of the integrated system; a full-width half maximum (FWHM) lateral resolution of 0.98 μm was confirmed. Finally, a variety of samples were imaged to verify the system’s practical use in an optical inspection system.

## Results

### Design of the multi-optical probe confocal imaging system with an increased working distance

**Figure 1:**
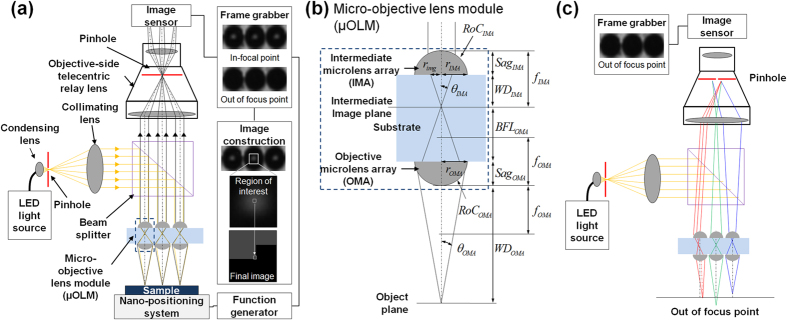
Schematic diagrams of the multi-optical probe confocal imaging system with increased working distance. **(a)** the proposed multi-optical probe confocal imaging system using the micro-objective lens module (μOLM). Example images from the image sensor with a relay lens aperture diameter of 1.5 mm. **(b)** Conceptual drawing of the micro-objective lens module composed of an objective microlens array (OMA) and the intermediate microlens array (IMA) and **(c)** the pinhole effect for blocking optical information from out of the focal point in the multi-optical probe confocal imaging system.

A μOLM composed of two microlens arrays, an objective-side telecentric relay lens, a light source, and an imaging sensor were applied to achieved the confocal imaging system. Figure 1(a) shows a schematic diagram of the designed optical system and an example of the image formation process. Generally, the working distance of an applied microlens is restricted to its focal length. Here, to increase the working distance of the objective microlens such that it exceeds the lens focal length, a μOLM was designed with two microlens arrays. The proposed design methodology using μOLM can provide a solution to make the working distance longer with considerably reduced loss of resolving power (see Supplementary Fig. S1). The module is composed of an OMA and IMA, as shown in Fig. 1(b). When the working distance (*WD*_*OMA*_) is greater than the focal length (*f*_*OMA*_) of the μOLM, the back focal length (*BFL*_*OMA*_) and convergence angle (*θ*_*IMA*_) can be calculated using Equations (1) and (2), which relate *f*_*OMA*_ and *WD*_*OMA*_, respectively:


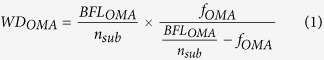



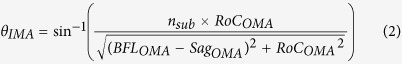



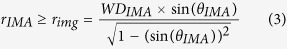



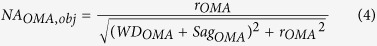


Here, *n*_*sub*_ is the refractive index of the substrate, and *Sag*_*OMA*_ and *RoC*_*OMA*_ are the sag height and radius of curvature, respectively. *BFL*_*OMA*_ shortens as *WD*_*OMA*_ lengthens. The transmission of optical information using a relay lens to the image sensor becomes more difficult because *θ*_*IMA*_ increases drastically, as indicated in Equation (2). An IMA was used to resolve this issue. The IMA converges the parallel beam from the light source to the intermediate image plane, and this beam is then focused to the object plane by the OMA. Subsequently, the beam is reflected by the sample and converges to the intermediate image plane due to the OMA. Finally, the beam is collimated by the IMA and propagated to the relay lens. To ensure that the collimated beam is delivered to the image sensor without a loss of optical information, the radius of the IMA, *r*_*IMA*_, was designed to be greater than the radius of the image circle generated by the OMA, *r*_*img*_, which can be calculated using Equation (3). The objective-side numerical aperture (NA) of the OMA (NA_OMA,obj_) was used to derive the optical performance of the designed μOLM with the increased working distance, as given in Equation (4).

The beam collimated by the IMA has a short propagating distance due to diffraction effects produced by the small radius of the microlens. This can cause a loss of optical information during transmission of the beam from the IMA to the image sensor. To solve this problem, an objective-side telecentric lens was applied as a relay optical system and was focused on the plane of the IMA. Additionally, the objective-side telecentric lens enables the system to secure sufficient space to ensure that the coaxial optical configuration is within the working distance of the relay lens. Moreover, due to the properties of a telecentric lens, the chief ray is parallel to the optical axis, and the optical signal collimated by the IMA passes the centre of the relay lens stop plane. Based on these properties, the aperture located on the relay lens stop plane can be used as the pinhole required in a confocal imaging system. A point light source is generated by the pinhole after the LED light source, and the aperture of the relay lens used as a pinhole for the confocal system is located in an optically conjugate plane in front of the detector. This configuration makes the presented system work as a confocal imaging system. As illustrated in Fig. 1(a,c), the optical information coming from the focal point of the optical axis of the OMA reaches the image sensor after passing through the centre of the relay lens aperture. In contrast, optical information out of the OMA focal point is blocked by the aperture. Therefore, a single aperture of the relay lens can be configured to act as a pinhole for the confocal imaging system that uses each microlens.

To confirm the feasibility of the proposed design for use in confocal imaging systems, a system was designed with a FOV of 1 mm × 1 mm, a resolution of 1 μm/pixel for the display circuit optical inspection system, and a working distance of 165.5 μm, which is greater than the possible particulate contamination size during the fabrication of a flat panel display. The μOLM was composed of an OMA and an IMA integrated on either side of a glass substrate (thickness: 320 μm). The geometrical specifications of the microlens arrays were as follows: the radii of the lens arrays were both 40 μm; the sag height of the OMA and IMA were 17.50 μm and 20.16 μm, respectively; the focal lengths of each microlens array were 81 μm and 74 μm, respectively, using a photoresist with a refractive index of 1.6724 at a wavelength of 572 nm; and the dimension of the μOLM array was 10 × 10 with a pitch of 100 μm. This configuration enables 1 mm × 1 mm size imaging with a scanning range of 100 μm × 100 μm. The working distance of the designed μOLM was 165.5 μm with an objective-side numerical aperture (NA) on the OMA, *NA*_*OMA,obj*_, and a value of 0.21, as given by Equations (1) and (4), respectively. The *r*_*img*_ of the designed system was calculated to be 16.22 μm. To ensure that the system is capable of transmitting an intermediate image on the OMA to the relay lens without any loss of optical information, *r*_*IMA*_ was designed with a value of 40 μm.

### Construction of the confocal imaging system with micro-objective lens module fabrication

**Figure 2:**
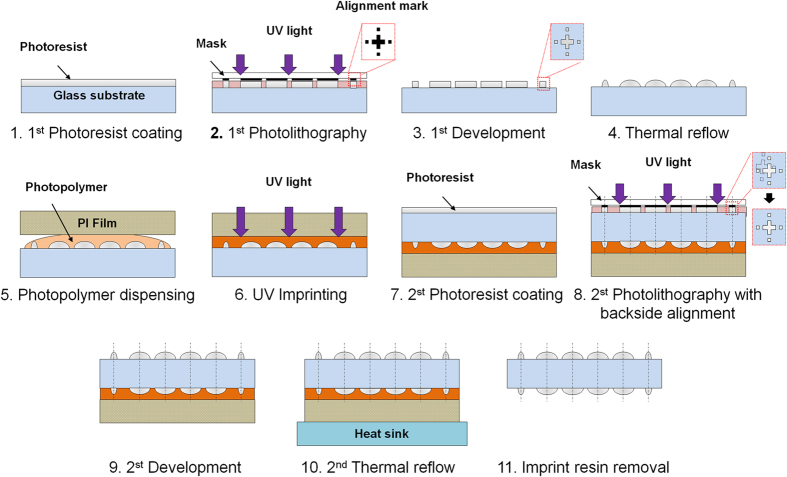
Schematic diagram of the μOLM fabrication process. The designed μOLM with two microlens arrays was fabricated using two steps of a photolithography process and a thermal reflow process. The OMA was fabricated preferentially, and the IMA was then fabricated. The UV imprint process was conducted to fabricate the OMA protection layer, which prevented damage during the additional process.

**Figure 3:**
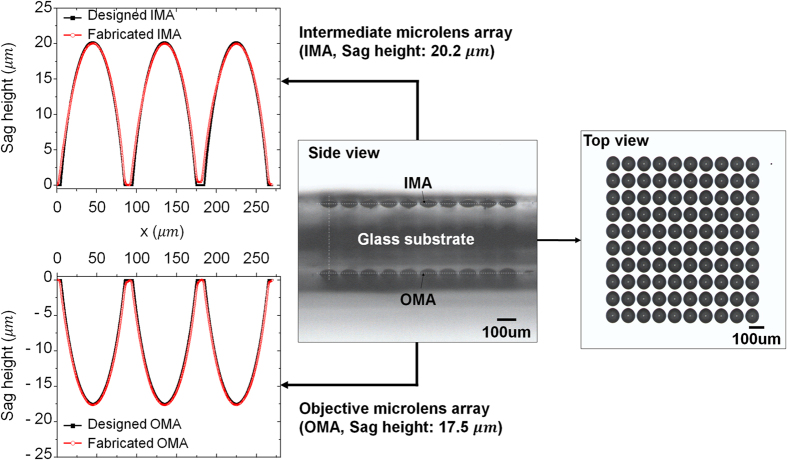
The μOLM sample image with profile measurement results. (left) sag height of the OMA and IMA was 17.5 μm and 20.2 μm. The geometrical deviations of fabricated microlens arrays from the designed value were less than 0.9%, (centre) the alignment deviation between the central axis of microlenses on either side were measured at 3.1 μm, and (right) image of the fabrication result on IMA side view showed the dimension of the μOLM is an array size of 10 × 10.

The designed μOLM with two microlens arrays was fabricated using two steps of a photolithography process and a thermal reflow process. Figure 2 shows the fabrication process of the μOLM with the microlens arrays. The sag height of the OMA and IMA were 17.5 μm and 20.2 μm, respectively. Figure 3 shows the fabricated μOLM and its profiles. The microlens array profiles were measured using a surface profiler (Form Talysurf PGI 840, Taylor Hopson, UK). The results confirmed that the geometrical deviations of the fabricated microlens arrays from the designed value were less than 0.9%. Furthermore, the alignment deviations between the central axis of microlenses on either side were measured to be 3.1 μm using a tool maker’s microscope (STM6, Olympus, Japan) at 10× magnification.

A 90-W white light light-emitting diode (LED) (WS-90, HEAAN, Korea) was selected. Although a single wavelength light source, such as laser, can achieve a greater resolving power, there are limitations in the simultaneous inspection of materials with different reflectances. A non-polarizing beamsplitter (10 mm VIS, Edmund Optics, USA) was used to implement the coaxial optical system between the light source and image sensor. A 1.4-megapixel charge-coupled device (CCD) image sensor (CM-140MCL, JAI, Japan) was used as the image sensor for data acquisition. An objective-side telecentric lens (KS_12K_TX35, Kisoo Precision, Korea) at 3.5× magnification, a working distance of 40 mm, and a FOV of 21 mm was used as a relay optical system equipped with a replaceable aperture. A nano-positioning system (P-561.3CD, PI, Germany) was used for sample scanning.

Raw images were captured using in-house synchronizing software encoded using LabView (National Instruments, USA). The intensities of the optical probes at the image plane of the telecentric lens were detected by the CCD, and the value from one CCD pixel was stored as a single pixel for the image. The rotational misalignment of the optical probe array and the translational direction of the scanning in the XY-direction, which can induce the image disconnection, were adjusted using a manual precision rotational stage^35^.

### Analysis of the optical performance of the constructed confocal imaging system

Because the resolution limit of the confocal imaging system was 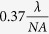 36,37, the theoretical optical resolution of the developed system can be obtained using Equation (5):


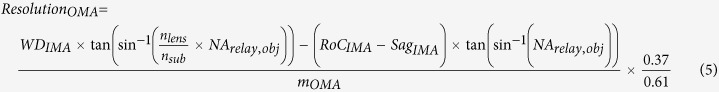


where *RoC*_*IMA*_ is the radius of curvature of the IMA, *m*_*OMA*_ is the magnification of the OMA, and *NA*_*relay, obj*_ is the objective-side NA of the relay lens.

The resolution increases as the aperture diameter of the relay optical system decreases, as given by Equation (5). However, as the aperture diameter decreases, the light intensity reaching the CCD is reduced in proportion to the square of the aperture’s radius. In this study, imaging tests were performed with an aperture diameter as small as 1.5 mm due to the limitations of the CCD sensitivity. To analyse the effects of the aperture diameter on the system resolution, imaging tests were completed using four different aperture diameters.

**Figure 4:**
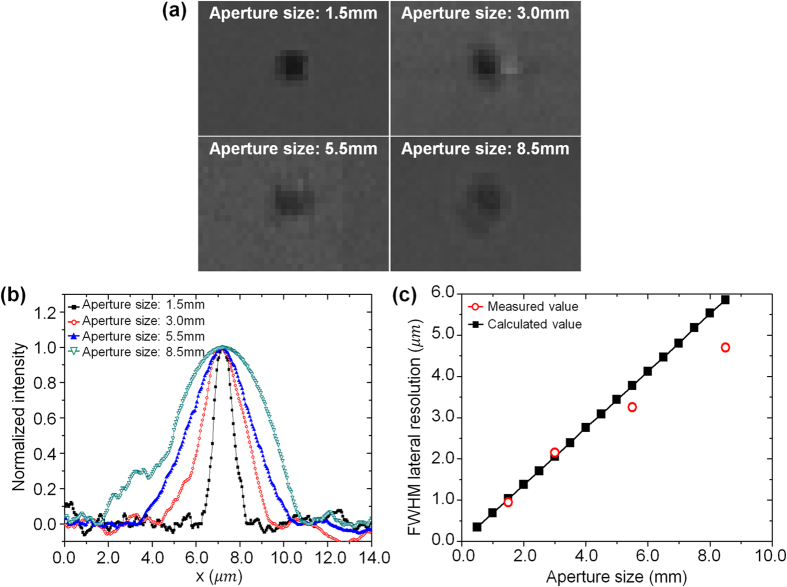
Analysis of the effect of aperture diameter on the lateral resolution. **(a)** sample images from a 4 μm × 4 μm square island pattern; **(b)** first-order derivatives of the normalized intensity; and **(c)** full width-half maximum (FWHM) measured resolution compared with the calculated values for various aperture diameters ranging from 1.5 mm to 8.5 mm. The maximum resolving power was 0.98 μm at a 1.5-mm aperture diameter.

Figure 4(a) compares the sample images with a 4 μm × 4 μm square island pattern for each aperture diameter. By comparing the images, it is possible to confirm intuitively that the resolution improves as the aperture diameter decreases. A knife-edge detection method was used to quantitatively evaluate the improved lateral resolution with a decreasing aperture diameter of the system. The image used for the knife-edge detection method was acquired using a sampling period of 50 nm. Figure 4(b) shows the first-order derivatives of the normalized intensity with a decreasing aperture diameter. The measured resolutions in the FWHM were compared with the calculated values shown in Fig. 4(c). The maximum resolving power was 0.98 μm at a 1.5-mm aperture diameter with a *NA*_*relay, obj*_ of 0.02. Using Equation (5), the calculated resolving power of the μOLM with two microlens arrays was 0.94 μm, which shows a good agreement with the measured value. It should be noted that the developed system using the μOLM with two microlens arrays improved the resolving power compared with the system using a single microlens array at the same working distance. The resolving power of a single microlens array with a focal length of 165.5 μm can be determined to be 2.00 μm using equation (SE1) in the supplementary information.

**Figure 5:**
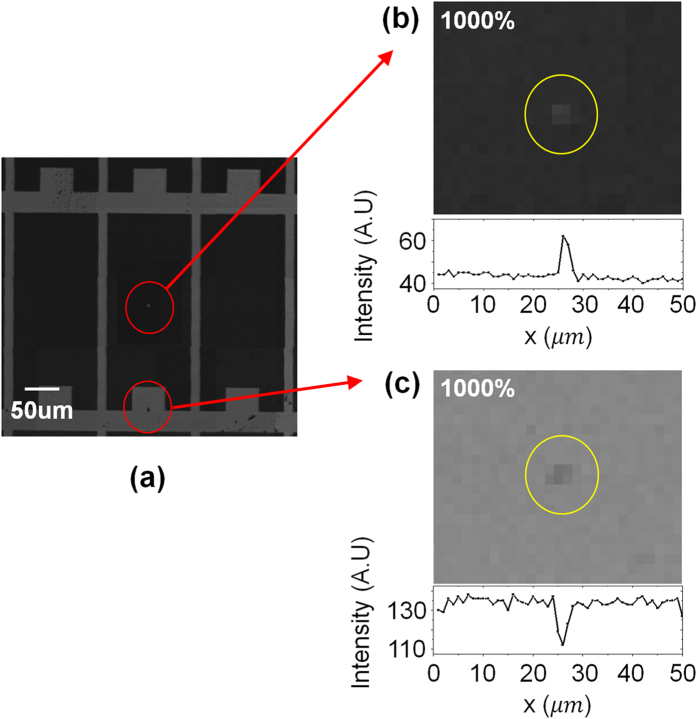
Images of a chrome-deposited photomask with a 1 μm × 1 μm island and pinhole pattern. **(a)** test panel image, **(b)** 1-μm island image, and **(c)** 1-μm pinhole image using the proposed confocal imaging system. The patterns are intuitively distinguished using both the 1000% magnified images and the intensity profiles.

To assess the system’s limitations in terms of resolving power, a chrome-deposited photomask with a 1 μm × 1 μm island and a pinhole pattern were imaged, as shown in Fig. 5. It should be noted that limitations in resolving power can be enhanced using a smaller aperture diameter and using a CCD with a greater sensitivity.

**Figure 6:**
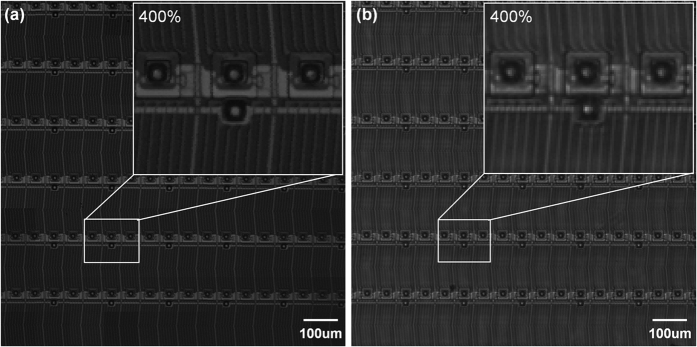
Thin-film transistor (TFT) pixel glass image. obtained using **(a)** the proposed confocal imaging system, and **(b)** a conventional inspection system with 400% magnification at a 1.5-mm aperture diameter. The confocal imaging system has a sharper edge on the micro-scale lines and structures due to the improved resolving power.

To determine whether the proposed imaging system could be applied to the inspection of an actual electrical circuit, a thin film transistor (TFT) pixel glass was imaged. Figure 6(a) shows the imaging results of the 1 mm × 1 mm FOV at 400% magnification obtained using the proposed confocal imaging system. A comparison with the same objective image taken using a conventional inspection system with a magnification of 5.0, NA of 0.12, 10 mm WD with coaxial light and a FOV of 1.4 mm × 1.0 mm (Kisoo Precision, Korea) confirmed that the confocal imaging system improved the resolving power (Fig. 6(b)). (see Supplementary Fig. S2~S4 for more examples).

## Discussion

This study developed a design methodology for a multi-optical probe confocal imaging system using an OMA that can be applied to a large area and allows high-resolution inspection. A system was constructed to verify the feasibility of the design, and tests were conducted to evaluate the proposed system’s optical performance. The proposed design methodology for the confocal imaging system overcomes the limitations of the short working distances typical of microlens arrays by incorporating a μOLM with two microlens arrays and a telecentric relay optical system. The design allows simplified, scalable integration by utilizing the aperture of the objective-side telecentric relay lens as a pinhole. This system has the advantages of reduced unit cost for optical system integration and simplified scaling for large-area measurements.

The μOLM was fabricated using backside alignment photolithography and a thermal reflow processes. The working distance of the module was 165.5 μm. A spherical microlens with a geometrical deviation of less than 0.9% compared with the designed lens was achieved. The resolution of the proposed confocal imaging system was evaluated using the knife-edge detection method with respect to the aperture diameter. The results confirmed that the FWHM lateral resolution was 0.98 μm with a 1.5-mm aperture diameter. Furthermore, the relationship between the FWHM lateral resolution and the aperture diameter confirmed that the system could achieve a greater resolution using a smaller aperture and a CCD with greater sensitivity. Finally, an imaging test of a TFT pixel glass with a 1 mm × 1 mm FOV was performed; the results confirmed that the proposed confocal imaging system could be exploited to inspect actual electronic circuits.

This study developed an alternative design methodology capable of producing a cost-effective inspection system with high optical resolution. The system overcomes the resolution limit of existing optical inspection systems. The implementation of a metre-scale inspection system, through system modulation based on the proposed design methodology and its parallelization, is a subject of on-going research.

## Methods

### Fabrication of μOLM

The designed μOLM with two microlens arrays was fabricated using a photolithography process and a thermal reflow process. Positive photoresist (AZ 9260, AZ Electronic Materials, USA) was spin-coated at 3,000 rpm on a glass substrate with a thickness of 320 μm (borosilicate glass, Schott, Germany) for 60 sec and baked on a hotplate at 110 °C for 120 sec. Micro-cylindrical pedestals with a thickness of 10.7 μm were then fabricated via photolithography using a quartz mask and mask aligner (MA-6, SUSS MicroTec, Germany). Here, the alignment marks of the mask were located on the four corners of the microlens array for a backside optical alignment process. The minimum line width of the alignment mark was designed to be 5 μm. I-line UV light was used at 1,250 mJ/cm^2^ and the substrate was immersed in positive developer (AZ 400 k, AZ Electronic Materials, USA) for 180 sec. After rinsing and drying, the prepared micro-cylindrical pedestals were processed at a thermal reflow of 160 °C for 25 sec to obtain a spherical lens shape.

A protective layer used to integrate the IMA on the back side of the OMA was fabricated with a polyimide (PI) film. A silicon urethane acrylate-based ultraviolet (UV)-curable photopolymer (MINS-ERM, Minuta Technology, Korea), which has a low surface energy to simplify the removal of the protective layer after fabrication of the IMA, was used as lens-shape conservation layer. The resin was then coated onto the OMA by applying a PI film and was polymerized using 3,500 mJ/cm^2^ broadband UV light exposure. The IMA was fabricated using the same photoresist coated at 1,500 rpm for 60 sec with an initial thickness of 12.6 μm after the same soft-baking process. The central axis for each microlens was aligned using an optical backside alignment process, and the micro-cylindrical pedestals were fabricated using the same development process as described above with 1,450 mJ/cm^2^ UV light exposure. The final thermal reflow process proceeded with a metal heat sink created on the protective layer under the same conditions as the prior thermal reflow process. Finally, the protective layer was removed.

## Additional Information

**How to cite this article**: Choi, W. *et al*. Design methodology for a confocal imaging system using an objective microlens array with an increased working distance. *Sci. Rep.* **6**, 33278; doi: 10.1038/srep33278 (2016).

## Supplementary Material

Supplementary Information

## References

[b1] Geissler M. & Xia Y. N. Patterning: Principles and some new developments. Adv. Mater. 16, 1249–1269 (2004).

[b2] Gates B. D. . New approaches to nanofabrication: Molding, printing, and other techniques. Chem. Rev. 105, 1171–1196 (2005). 15826012 10.1021/cr030076o

[b3] Biswas A. . Advances in top-down and bottom-up surface nanofabrication: techniques, applications & future prospects. Adv. Colloid Interface Sci. 170, 2–27 (2012). 22154364 10.1016/j.cis.2011.11.001

[b4] Shim W. . Hard-tip, soft-spring lithography. Nature 469, 516–521 (2011). 21270890 10.1038/nature09697

[b5] Wang M. . CVD growth of large area smooth-edged graphene nanomesh by nanosphere lithography. Sci. Rep. 3, 1238 (2013). 23393620 10.1038/srep01238 PMC3566595

[b6] Lin H. C. . Deep ultraviolet laser direct write for patterning sol-gel InGaZnO semiconducting micro/nanowires and improving field-effect mobility. Sci. Rep. 5, 10490 (2015). 26014902 10.1038/srep10490 PMC4444848

[b7] Qin D., Xia Y. & Whitesides G. M. Soft lithography for micro-and nanoscale patterning. Nat. protoc. 5, 491–502 (2010). 20203666 10.1038/nprot.2009.234

[b8] Chang C. & Sakdinawat A. Ultra-high aspect ratio high-resolution nanofabrication for hard X-ray diffractive optics. Nat. Commun. 5, 4243, (2014). 24970569 10.1038/ncomms5243

[b9] Rajasekharan R. . Filling schemes at submicron scale: Development of submicron sized plasmonic colour filters. Sci. Rep. 4, 6435 (2014). 25242695 10.1038/srep06435 PMC4170198

[b10] Li C., Zhao L. R., Mao Y. F., Wu W. G. & Xu J. Focused-ion-beam induced Rayleigh-Plateau instability for diversiform suspended nanostructure fabrication. Sci. Rep. 5, 8236 (2015). 25649055 10.1038/srep08236 PMC4650821

[b11] Nomura K. . Room-temperature fabrication of transparent flexible thin-film transistors using amorphous oxide semiconductors. Nature 432, 488–492 (2004). 15565150 10.1038/nature03090

[b12] Lim J., Gruner P., Konrad M. & Baret J.-C. Micro-optical lens array for fluorescence detection in droplet-based microfluidics. Lab Chip 13, 1472–1475 (2013). 23455606 10.1039/c3lc41329b PMC3697795

[b13] Kang S. Micro/Nano Replication: Processes and Applications 7–14, 31–42 (John Wiley & Sons, 2012).

[b14] Kim J.-H., Ahn S., Jeon J. W. & Byun J.-E. A high-speed high-resolution vision system for the inspection of TFT LCD in Industrial Electronics, *2001*. Proceedings. ISIE 2001. IEEE International Symposium on 101–105 (2001).

[b15] Malamas E. N., Petrakis E. G., Zervakis M., Petit L. & Legat J.-D. A survey on industrial vision systems, applications and tools. Image Vis. Comput. 21 171–188 (2003).

[b16] Golnabi H. & Asadpour A. Design and application of industrial machine vision systems. Robot. Comput.-Integr. Manuf. 23, 630–637 (2007).

[b17] Perng D.-B., Liu H.-W. & Chang C.-C. Automated SMD LED inspection using machine vision. Int. J. Adv. Manuf. Technol. 57, 1065–1077 (2011).

[b18] Koenig J. G. Display component quality and process control with advanced automated optical inspection. In SID Int. Symp. Dig. Tec. 1140–1143 (2013).

[b19] Chang M., Chou Y., Lin P. & Gabayno J. Fast and high-resolution optical inspection system for in-line detection and labeling of surface defects. CMC-Comput. Mat. Contin. 42, 125–140 (2014).

[b20] Huang S.-H. & Pan Y.-C. Automated visual inspection in the semiconductor industry: A survey. Comput. Ind. 66, 1–10 (2015).

[b21] Ueda N. . An ultra high density 736‐ppi liquid crystal display using InGaZnO platform. In SID Int. Symp. Dig. Tec. 931–934 (2015).

[b22] Takubo Y., Hisatake Y., Lizuka T. & Kawamura T. Ultra‐high resolution mobile displays. In SID Int. Symp. Dig. Tec. 869–872 (2012).

[b23] Lu R. S., Shi Y. Q., Li Q. & Yu Q. P. AOI techniques for surface defect inspection. Appl. mech. Mater. 36, 297–302 (2010).

[b24] Webb R. H. Confocal optical microscopy. Rep. Prog. Phys. 59, 427–471 (1996).

[b25] Uhlmann E., Oberschmidt D. & Kunath-Fandrei G. 3D-analysis of microstructures with confocal laser scanning microscopy. In *Proc. ASPE winter topical meeting–Machines and processes for micro-scale and meso-scale fabrication, metrology and assembly, ASPE, Gainesville*. 93–97 (2003).

[b26] Garini Y., Vermolen B. J. & Young I. T. From micro to nano: recent advances in high-resolution microscopy. Curr. Opin. Biotechnol. 16, 3–12 (2005) 15722009 10.1016/j.copbio.2005.01.003

[b27] Egner A., Andresen V. & Hell S. W. Comparison of the axial resolution of practical Nipkow-disk confocal fluorescence microscopy with that of multifocal multiphoton microscopy: theory and experiment. J. Microsc. 206, 24–32 (2002). 12000560 10.1046/j.1365-2818.2002.01001.x

[b28] Lim J., Jung M., Joo C. & Kang S. Development of micro-objective lens array for large field-of-view multi-optical probe confocal microscopy. J. Micromech. Microeng. 23, 065028 (2013).

[b29] Tanaami T. . High-speed 1-frame/ms scanning confocal microscope with a microlens and Nipkow disks. Appl. Optics 41, 4704–4708 (2002). 10.1364/ao.41.004704 12153106

[b30] Toshiyoshi H., Su G.-D. J., LaCosse J. & Wu M. C. A Surface micromachined optical scanner array using photoresist lenses fabricated by a thermal reflow process. J. Lightwave Technol. 21, 1700–1708 (2003).

[b31] Biryukov S., Faiman D. & Goldfeld A. An optical system for the quantitative study of particulate contamination on solar collector surfaces. Sol. Energy 66, 371–378 (1999).

[b32] Cooper D. W. Particulate contamination and microelectronics manufacturing - an introduction. Aerosol Sci. Technol. 5, 287–299 (1986).

[b33] Junge C. The size distribution and aging of natural aerosols as determined from electrical and optical data on the atmosphere. J. Meteorol. 12, 13–25 (1955).

[b34] Kuhlbusch T. A., Neumann S. & Fissan H. Number size distribution, mass concentration, and particle composition of PM1, PM2.5, and PM10 in bag filling areas of carbon black production. J. Occup. Environ. Hyg. 1, 660–671 (2004). 15631057 10.1080/15459620490502242

[b35] Lim J., Jung M., Hwang S. Y. & Kang S. Development of optical system with rotational misalignment adjustment for multi-optical-probe confocal microscopy. J. Vac. Sci. Technol. B 30, 06F702 (2012).

[b36] Kino G. S. & Corle T. R. In Confocal scanning optical microscopy and related imaging systems 31–40 (Academic Press, 1996).

[b37] Park J. S., Choi C. K. & Kihm K. D. Optically sliced micro-PIV using confocal laser scanning microscopy (CLSM). Exp. Fluids 37, 105–119 (2004).

