# Supplementary material for: Design methodology for a confocal imaging system using an objective microlens array with an increased working distance
Source: Sci Rep. 2016 Sep 12;6:33278. doi: 10.1038/srep33278 (PMC5018843; doi:10.1038/srep33278)
Supplement: Supplementary Information [file srep33278-s1.doc]

**Supplementary Information**

**Design methodology for a confocal imaging system using an objective microlens array with an increased working distance**

# Woojae Choi^1,+^, Ryung Shin^1,2,+^, Jiseok Lim^2,3,*^, and Shinill Kang^1,2,**^

^1^School of Mechanical Engineering, Yonsei University, Seoul, 03722, South Korea.

^2^National Center for Optically-assisted Ultrahigh-precision Mechanical Systems, Yonsei University, Seoul, 03722, South Korea.

^3^School of Mechanical Engineering, Yeungnam University, Gyeongsan*,* 38541, South Korea

*jlim@yu.ac.kr.

**snlkang@yonsei.ac.kr.

+these authors contributed equally to this work.

# Comparison of the calculated resolving power of an SMA-based system

# and the developed system

The theoretical optical resolution of a system using a single microlens array (SMA) as the objective lens can be calculated using equation (SE1):

$Resoulution_{SMA}=WD_{SMA}\times(\sin^{-1}(NA_{relay,obj}))$ (SE1)

where *WD_SMA_* is the working distance of the SMA and *NA_relay,obj_* is the objective-side numerical aperture (NA) value of the telecentric lens.

Figure S1 compares the resolving power of an SMA-based system calculated using equation (SE1) and the developed system calculated using equation (5). As shown in Fig. S1, the resolving power of the proposed system is less sensitive to the working distance than that of the SMA-based system.

**Figure S1.** Changes in the resolving power with the working distance.

# 2. Imaging test results of actual electrical circuit samples

Sample images were obtained via the image-acquisition process using the synchronising software and an image-construction program. Raw images were captured at a sampling rate of 1 μm/frame. The FOV was 1 × 1 mm, and the working distance of the system was 165.5 μm.

Figure S2 shows the imaging results of the colour filter pixel for a thin-film transistor (TFT) with a 4× magnified image in the inset. The TFT data line image results are shown in Fig. S3. Figure S4 shows the imaging results of the organic light-emitting diode (OLED) pixel glass with a 1 × 1 mm field of view (FOV) at the same magnification as in the previous sample image. The proposed confocal imaging system had an improved resolving power based on comparison of the imaging examples.


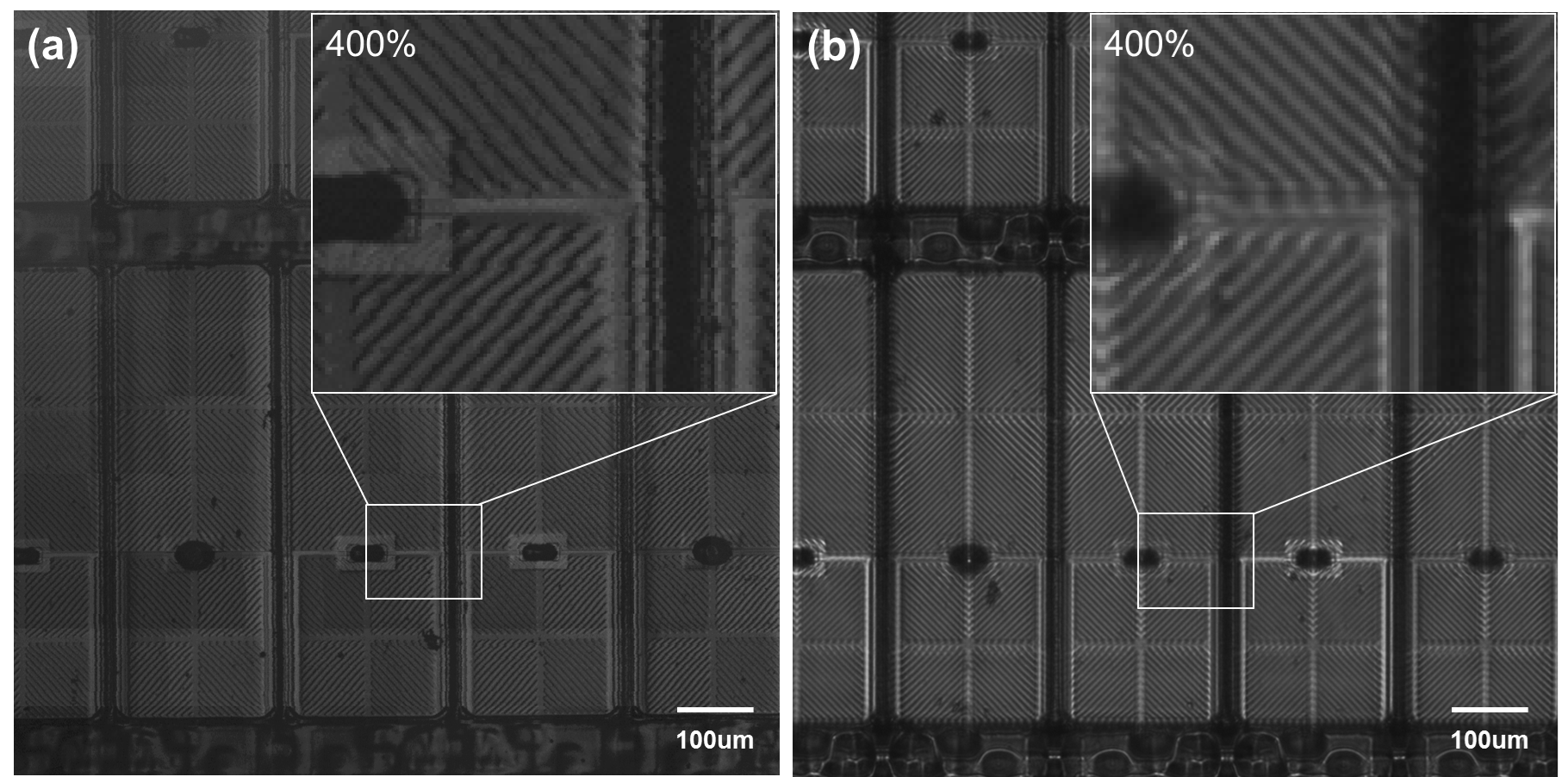


**Figure S2.** **Thin-film transistor (TFT) color filter pixel:** obtained via **(a)** the proposed confocal imaging system and **(b)** a conventional inspection system with 400 % magnification at a 1.5-mm aperture diameter.


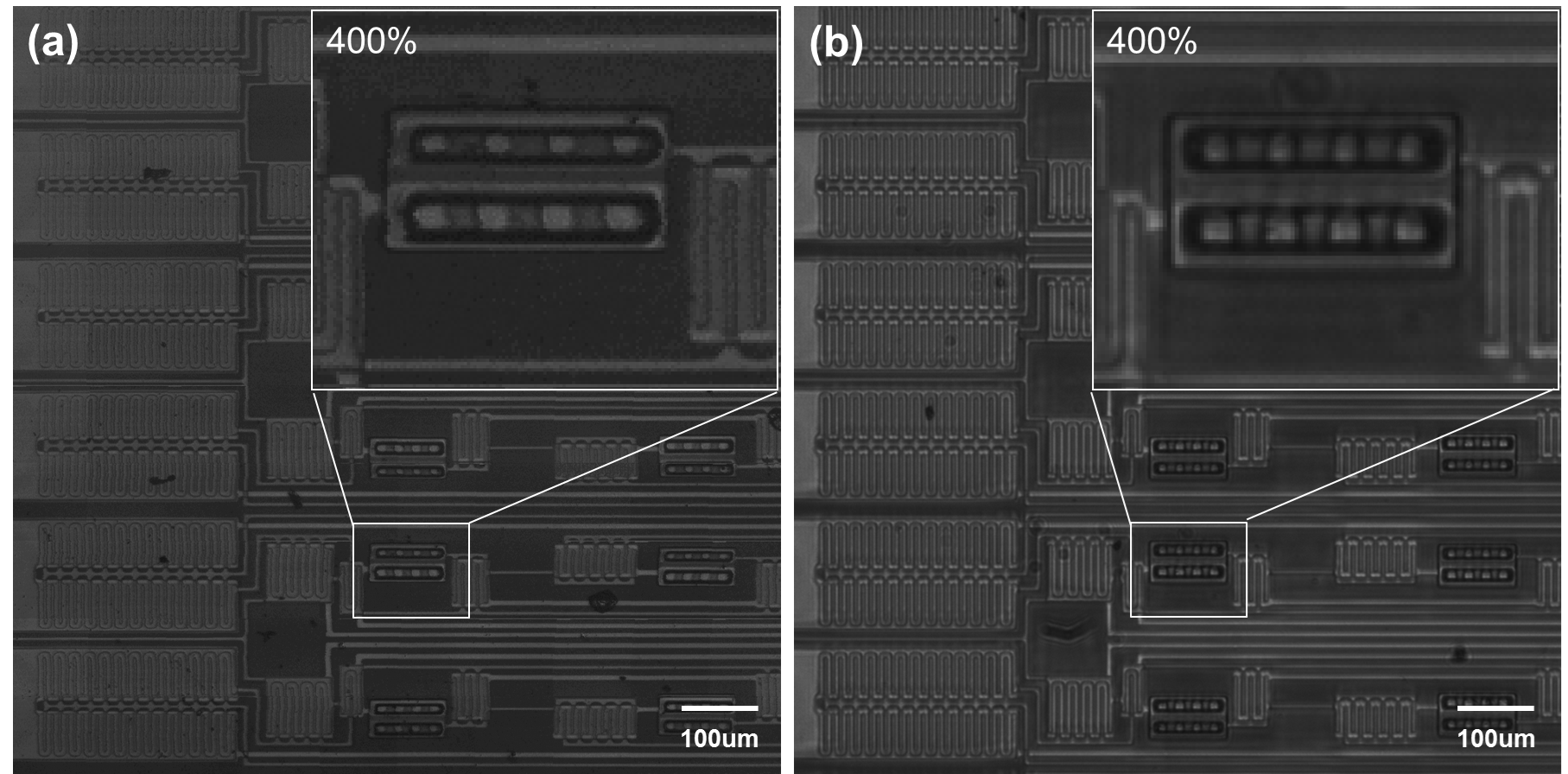


**Figure S3.** **TFT data line image:** obtained via **(a)** the proposed confocal imaging system and **(b)** a conventional inspection system with 400 % magnification at a 1.5-mm aperture diameter.


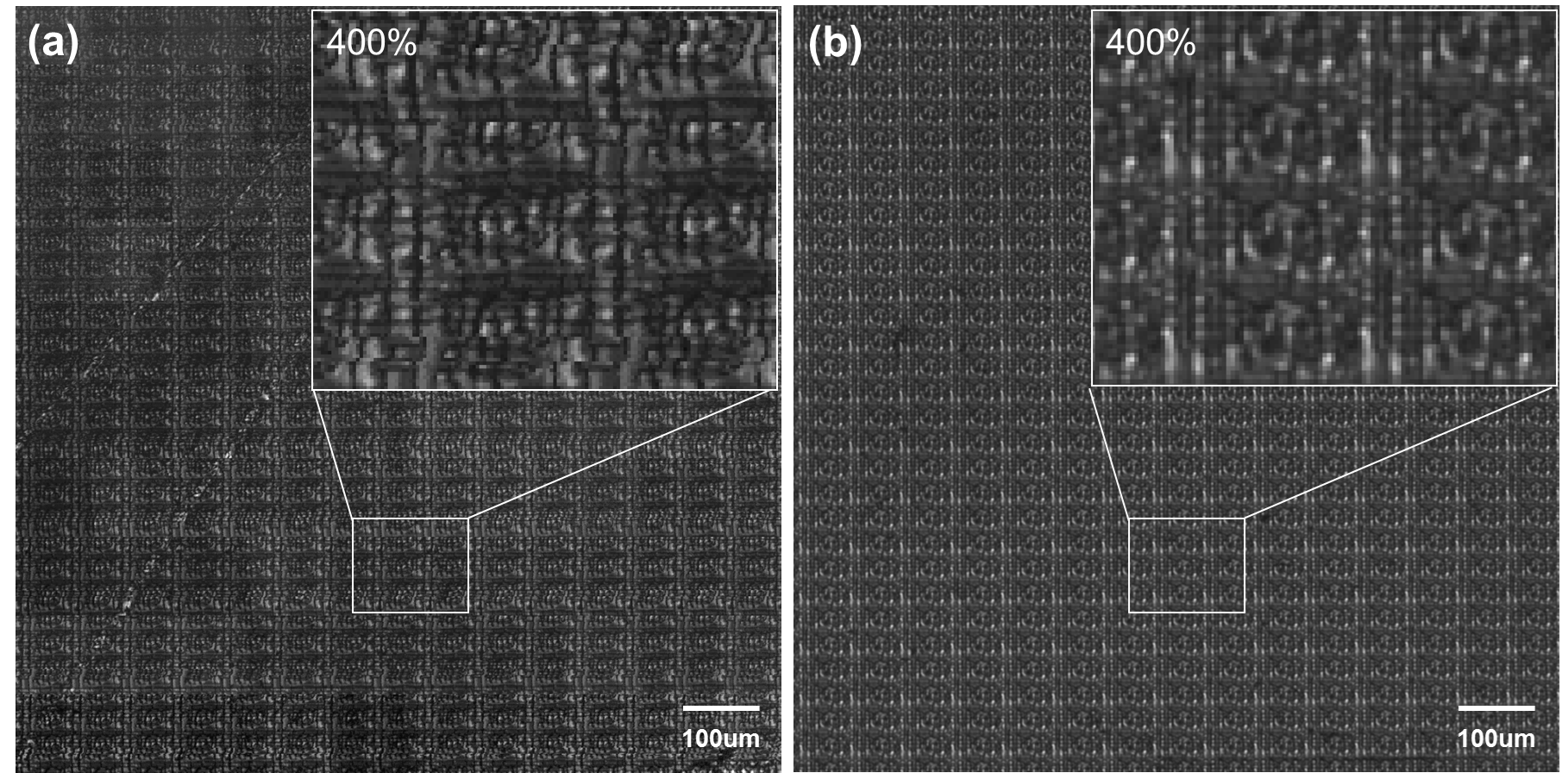


**Figure S4. OLED pixel glass image:** obtained via **(a)** the proposed confocal imaging system and **(b)** a conventional inspection system with 400 % magnification at a 1.5-mm aperture diameter.
